# Low levels of Human Cytomegalovirus Infection in Glioblastoma multiforme associates with patient survival; -a case-control study

**DOI:** 10.1186/2042-4280-3-3

**Published:** 2012-03-16

**Authors:** Afsar Rahbar, Giuseppe Stragliotto, Abiel Orrego, Inti Peredo, Chato Taher, Jan Willems, Cecilia Söderberg-Naucler

**Affiliations:** 1 Department of Medicine, Solna, Center for Molecular Medicine, Karolinska Institutet, 171 76 Stockholm, Sweden; 2 Department of Neurology , Karolinska University Hospital, Stockholm, Sweden; 3 Department of Pathology, Karolinska University Hospital, Stockholm, Sweden; 4 Department of Neurosurgery, Karolinska University Hospital, Stockholm, Sweden

**Keywords:** HCMV, Glioblastoma, Survival

## Abstract

**Background:**

Glioblastoma multiforme (GBM) represent the most aggressive brain tumor with a median overall survival of about 12-15 months. Over 90% of GBM tumors have recently been shown to be infected with human cytomegalovirus (HCMV). In this case-control study, we evaluated whether there was an association between the grade of HCMV infection and long-term survival (> 18 months) in GBM patients.

**Material and methods:**

Brain tumor tissue sections from consecutive GBMs patients who survived more than 18 months (n = 40), and an equal number of GBM patients, matched to date of diagnosis and surgery, operated at Karolinska University Hospital in 2000-2005 were selected. HCMV infection grade was determined by estimation of the number of HCMV positive cells (scored negative or grade 1-4) in tumor tissue specimens. Using Chi-Square test and logistic regression analysis, we analyzed whether there was an association between long-term survival and HCMV low-grade infection or other clinical parameters known to be associated with prolonged survival of GBM patients; age under 50 years, radical surgery or low recursive partition analysis (RPA) subclass.

**Results:**

HCMV infection was detected in tumor samples from 79 of 80 patients (99%). Among patients surviving > 18 months, HCMV infection grade 1 in the GBM tumor was predominant. A low grade HCMV infection was found in 19 patients, of these 16 survived > 18 months. Thus, 16 of 40 (40%) GBM patients who lived > 18 months had low-grade HCMV infection while only 3 of 40 (8%) GBM patients who lived < 18 months did (*p .0006*, *Chi-Square test*). Multiple logistic regression analyses yielded an odds ratio estimate of 6.604 with 95% confidence interval (1.36-32.1) (*p .019*) for low grade HCMV after adjustment for RPA class III and IV, radical surgery, age and gamma knife treatment.

**Conclusion:**

In conclusion, we found that low-grade HCMV infection was strongly associated with long-term survival in GBM patients.

## Introduction

Glioblastoma multiforme grade IV (GBM) is the most aggressive and common brain tumor in humans. In Sweden, with a population of 9 million, there are approximately 300 new GBM cases per year [1]. Histologically, GBM is characterized by the presence of necrotic areas in the brain tissue surrounded by anaplastic cells and hyperplastic blood vessels, and a disparate genetic signature, which illustrates the heterogeneity of this tumor [2,3]. Current treatments such as surgical resection, radiation, and chemotherapy are relatively ineffective due to the aggressive nature of GBM. Despite increasing molecular knowledge of GBM tumors, few new therapeutic strategies have been offered these patients during the past decades and these patients still have a poor prognosis with a median survival of 12-15 months [4]. Recent studies have reported the presence of an active Human Cytomegalovirus (HCMV) infection in 90-100% of GBM tumors [5-7].

HCMV belongs to the *herpesviridea *family and maintains latency in pre-monocytic cells after a primary infection [8]. Viral reactivation can occur when latently infected monocytes undergo differentiation to macrophages or dendritic cells, which involves stimulation with inflammatory cytokines [9,10]. Thus, it is possible that the inflammatory environment in the glioblastoma tumor triggers reactivation of latent HCMV.

Although there is compelling evidence that the HCMV protein US28 may be oncogenic, this virus has not been considered to be oncogenic. Rather, HCMV proteins confer a variety of biological functions that potentially affect tumor biology and contribute to cancer progression. HCMV proteins for example interact with p53, Rb and PTEN and influence cell-cycle regulation, induce chromosomal aberrations, cellular differentiation, migration, angiogenesis and confer immune evasion mechanisms [11-16]. Recent studies have demonstrated that HCMV-US28 induces cyclo-oxygenase-2 (COX2) expression, production of vascular endothelial growth factor (VEGF) and tumor formation in vivo, via activation of nuclear factor kappa B (NF-kB), STAT3 phosphorylation and accumulation of beta cathenin in the cell nucleus. Furthermore, we found that phosphorylated STAT-3 was correlated with poor survival of GBM patients [17-19]. Thus, this virus may either be an epiphenomenon of glioblastomas or may through its sophisticated strategies that affect tumor biology and provide immune evasion strategies contribute to cancer progression. We recently demonstrated that targeting HCMV infection in medulloblastoma tumors with an anti-viral drug and a COX-2 inhibitor prevented tumor growth by 72% in an animal model [20]. These observations suggest that HCMV may contribute to the tumorgenesis of medulloblatsoma tumors, and that the virus may be a novel target for cancer therapy. We hypothesized that if the virus affects tumor progression, a low grade HCMV infection in glioblastomas may be associated with longer patient survival. In this case-control study, we investigated if the grade of HCMV infection in GBM tumors was associated with survival over 18 months.

## Materials and methods

### Patient samples

We evaluated the association between HCMV infection grades at diagnosis and survival time in a case-control cohort of patients with GBMs operated at Karolinska University Hospital between 2000 and 2005. Paraffin embedded brain tumor samples from first surgery of all available consecutive patients who lived > 18 months were collected and similar number of samples obtained from GBM patients matched to date of first surgery and diagnosis were included (Table 1). All patients had World Health Organization (WHO) grade IV GBM and received standard treatment. At least two experienced neuropathologists (mainly AO and JW) independently confirmed the diagnosis. Classification of patients was performed according to the adapted European Organization for Research and Treatment of Cancer recursive partition analysis classification [21]. The study was approved by the Ethics Committee at Karolinska Institutet, Stockholm, Sweden (2008/628-31/2).

**Table 1:** Patients' characteristics

Characteristics	Case control cohorts	
	**Patients surviving**	**Patients surviving**

	**> 18 months (n = 40)**	**≤ 18 months (n = 40)**

	**(2000-2005)**	**(2000-2005)**

**Age (years)**		

**< 50**	12(30%)	9 (23%)

≥ **50**	28(70%)	31(77%)

**Gender**		

**Male**	24(60%)	24(60%)

**Female**	16(40%)	16(40%)

**RPA class**		

**III-IV**	28(70%)*	10(25%)*

**V-VI**	12(30%)	30(75%)

**Extent of surgery**		

**Partial**	3 (7.5%)	9 (22.5%)

**Radical**	37(92.5%)#	31(77.5%)#

**Gammaknife**		

**No**	28(70%)	35(87.5%)

**Yes**	12(30%)	5 (12.5%)

**RT + Temozolomide**		

**No**	32(80%)	39(97%)

**yes**	8 (2%)	1 (3%)

**RT + adjuvant chemotherapy**		

**No**	17(42%)	21(52%)

**Yes**	23(58%)	19(48%)

**Median follow-up time of those alive at study closure (months)**	48 (n = 15)	0 (n = 0)

A significant association was observed between RPA III-VI and number of patients with overall survival > 18 months *: *p < .0001*. No significant association was observed between radical surgery and longer patients survival #: *p .06 *(Chi-Square test)

### Immunohistochemistry and *in Situ Hybridization*

Paraffin embedded brain tumor tissue sections (6 μm) were analyzed by sensitive immunostaining as previously described [5]. Briefly, tissue sections were de-waxed in Xylene and rehydrated in alcohol series. After post fixation in 4% neutral buffered formalin (Pharmacy at Karolinska University Hospital, Sweden), sections were enzymatic treated by pepsin (Sigma-Aldrich AB, Stockholm, Sweden) in 3 min at 37°C and treated in Citra buffer pH 7.6 (Biogenex, San Ramon, CA). Endogenous peroxidase activity was blocked with 3% H_2_O_2_, biotin and avidin were blocked with the Biotin/Avidin Blocking kit (DakoCytomation, Denmark) and Fc receptors were blocked with Fc receptor blocker (Innovex Sciences, US). The following primary antibodies were used: anti-HCMV-IEA (reacts with an immediate early non-structural antigen of 68-72 kDa), HCMV late antigen (LA, reacts with a late protein of 47-55 kD) (both IgG2a, Chemicon International, US). Samples were stained with antibodies against smooth muscle cell alpha actin (IgG2a, Biogenex) or no primary antibody (only dilution buffer) served as controls. Colorimetric determination was performed with a three-step horseradish peroxidase detection system (BioGenex) with the chromogen diaminobenzidine (DAB) (Innovex Sciences, US). AR (scientist) and AO (neuropathologist) examined the specimens; neither had access to the clinical records of the patients at the time of grading.

HCMV Infection was graded according to the estimated percentage of infected cells in the specimen: negative or grade 1, < 25%; grade 2, ≥ 25% to 50%; grade 3, ≥ 50% to 75%; and grade 4, > 75%. Proliferation of tumor cells (MIB index, Ki-67), p53 mutation, mitosis, glial fibrillary acidic protein (GFAP), were detected with automated immunohistochemical staining protocols at our hospital.

Tissue sections were examined for HCMV-DNA by in situ hybridization protocols as described previously [5]. Briefly, slides were pretreated as described above for immunohistochemistry. After pretreatment in Citrate buffer, slides were de-hydrated in alcohol series and air dried before adding HCMV-DNA total genome fluorescein labeled probes (Zymed Labs, South San Francisco, CA). Alu DNA sequence specific for endogenous and negative (nonspecific DNA) fluorescein labeled probes were used as positive and negative probes, respectively, (both control probes were from Zymed Labs, South San Francisco, CA). Tissue sections were denatured at 90°C for 10 minutes and hybridized at 37°C over night using a Misha thermocycler (DakoCytomation, Denmark). Endogenous peroxidase activity and FC receptors were blocked as was described above for immunohistochemistry. Fluorescein labeled probe was detected by using mouse anti - fluorescein antibodies (BioSite, Stockholm, Sweden), horseradish peroxidase labeled goat anti mouse antibodies (BioGenex) and DAB (Innovex Sciences).

### Statistical analysis

All statistical analyses were performed by a medical statistician (Fredrik Hansson, Commitum, Lund, Sweden). Chi-square test and logistic regression analysis was used to assess the association between survival > 18 months and HCMV infection while adjusting for known prognostic factors such as age, RPA class and extent of resection (and use of gamma knife). Power calculations were used to estimate the number of patients that should be included in the study. Calculating with a power of 80% with an alpha level of 5% and a follow-up time of 3 years, using a formula from Lachin JM. [22], the required number of patients was 70. *P values < .05 *were considered significant.

## Results

### HCMV infection level is associated with overall survival in GBM patients

**Figure 1:**
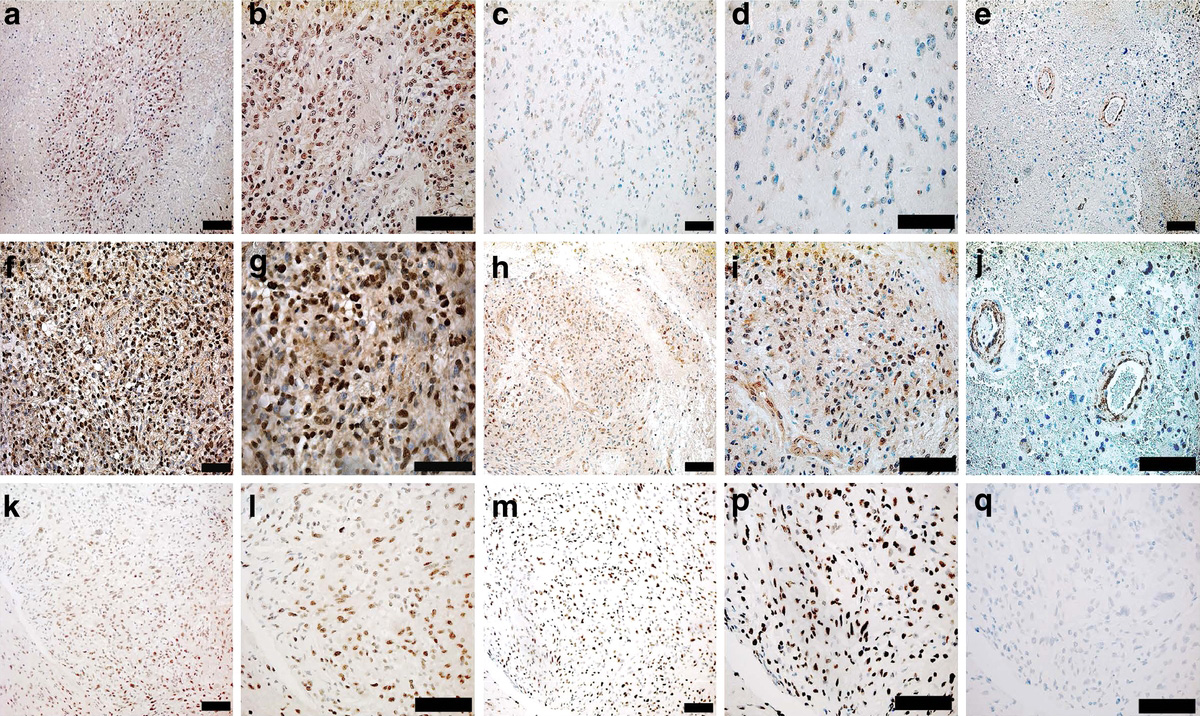
**Detection of HCMV expression by immunohistochemistry**. Low-grade HCMV-IEA infection (A, B), low-grade HCMV-LA infection (C, D) and SMC-alpha actin (E) in tissue sections obtained from a GBM patient. Moderate to high-grade HCMV-IEA (F, G), moderate to high grade HCMV-LA (H, I) and SMC-alpha actin (J) in GBM. Bar, 50 μm Detection of HCMV DNA by *in situ *hybridization (K, L), Alu; positive control (M, P) and negative control (Q) in GBMs. Bar, 50 μm.

HCMV-IEA was detected in 79 (99%) of 80 tumor samples obtained from GBM patients, and HCMV-LA in 76 (95%) of 80 samples. HCMV proteins were detected in tumor cells but not in surrounding non-tumor cells (Figure 1). HCMV infection was confirmed by *in situ *hybridization in 7 samples (one negative and 6 HCMV positive). HCMV DNA was present in all HCMV-positive samples (Figure 1) but not in the HCMV-negative sample.

HCMV infection grade was determined by estimation of the number of HCMV positive cells and scored as grade 1-4 depending on the estimated percentage of positive cells in tumor tissue specimens (grade 1; < 25%, grade 2; ≤ 25%-50%, grade 3; ≤ 50%-75% and grade 4; > 75%). To examine the influence of the HCMV infection grade on survival, two patient populations; long-term survivors (> 18 months (n = 40)) and an equal number of patients that survived ≤ 18 months (n = 40) matched to date of first surgery and diagnosis for HCMV infection grade (Table 1), were analyzed. In the cohort of patients who lived > 18 months, 40% of the patients were negative or had grade 1 HCMV-IE infection and median OS for these was 42.5 months (n = 16). 10% of the patients had grade 2 and Median OS was 25.5 months (n = 4), 15% of patients had grade 3 and median OS was 25 months (n = 6), and 35% of patients hade grade 4 and median OS was 26 months (n = 14). Thus, survival > 18 months does not clearly change as a function of grade; there seems to be a threshold level for the significance of HCMV for survival. It was due to this that the subsequent dichotomization to negative to grade 1 vs grade 2-4 were used. In the cohort of patients that survived ≤ 18 months; 8% and 20% had grade 1 and 2 HCMV-IE infection (n = 3 and n = 8) respectively while 25% and 47.5% had grade 3 and grade 4 infection (n = 10 and n = 19), respectively. In the cohort of GBM patients that survived > 18 months, 16 of 40 patients (40%) had low-grade HCMV-IEA infection. Among patients surviving ≤ 18 months only 3 of 40 (8%) had low-grade HCMV-IEA infection. Low-grade HCMV-IEA infection was strongly associated with survival > 18 months (*p *.0006). Low-grade HCMV-LA infection was detected in 19 of 40 (48%) tumors of patients that survived > 18 months and in 14 of 40 (35%) patients that survived ≤ 18 months (*p .26*).

We also observed a significant association between RPA subclass III-VI and overall survival > 18 months (*p < .0001*, Chi-Square test), but not for radical surgery (*p .06*, Chi-Square test), age, gender or treatment (Table 1). Next, we performed multiple logistic regression analysis in order to estimate odds ratio for survival > 18 months in patients with low-grade versus moderate to high grade-HCMV infection and for other prognostic factors important for survival of GBM patients (age, extent of resection, and RPA subclass). We found an odds ratio of 6.604 (*p .019*, 95% CI, 1.4 to 32.1) for low grade HCMV-IEA infection and odds ratio for RPA class III and IV was; 8.2 (*p .002*, 95% CI, 2.1 to 31) in patients with low-grade HCMV-IEA infection. We also found an odds ratio of 9.5 (*p. 0006*, 95%CI, 2.6 to 34.5) for RPA class III and IV in patients with low-grade HCMV-LA infection. However, we did not observe an association with extent of surgery, age, gender and gamma knife treatment for overall survival > 18 months (Table 2).

**Table 2:** Odds Ratio Estimates for patients surviving > 18 months in patients with low-grade versus high grade-HCMV infection in their tumors and prognostic factors important for GBM patients survival (Age, Extent of Resection, RPA Subclass and gamma knife treatment) by Multiple Logistic regression analysis.

Characteristic	HCMV-IEA					HCMV-LA		
	**95% LCL**	**Odds ratio**	**95% UCL**	***P value***	**95% LCL**	**Odds ratio**	**95% UCL**	***P value***

**HCMV infection: Non to Low-grade vs. moderate to high-grade**	1.359	6.604	32.094	.019*	0.425	1.264	3.758	*.67*

**Age group: **<**50 vs ≧ 50 y**	0.080	0.338	1.428	.*142*	0.081	0.339	1.412	*.137*

**Gender Female vs Male**	0.317	0.965	2.938	*.95*	0.335	0.962	2.763	*94*

**Extent of surgery: Radical vs partial**	0.404	2.489	15.348	*.326*	0.342	1.723	8.691	*.50*

**RPA: III and IV vsV and VI**	2.148	8.161	30.997	.002*	2.615	9.504	34.545	.0006*

**Gammaknife vs. no Gammaknife treatment**	0.326	1.339	5.505	.*068*	1.41	1.409	5.475	.*62*

*: considered significant, LCL (lower confidence interval), UCL (upper confidence interval)

No differences were observed in the number of patients with either no or low or moderate to high grade HCMV infection in regards to different therapies, except for gamma knife treatment (Table 3). In patients with no or low-grade HCMV-IEA infection, 7 (37%) of 19 patients had received gamma knife treatment (6 patients survived > 18 months, one patient survived ≤ 18 months) as compared to 10 (16.4%) of 61 patients with moderate to high-grade HCMV-IEA infection (6 patients survived > 18 months, 4 patients survived ≤ 18 months, Table 3).

**Table 3:** Therapy in patients whose tumors had low and high-grade infection with human cytomegalovirus Immediate-early (IEA) and late antigen (LA)

Therapy	Low-grade HCMV-IEA (n = 19)	High-grade HCMV-IEA (n = 61)	Low-grade HCMV-LA (n = 33)	High-grade HCMV-LA (n = 47)
Gamma knife	7 (37%)	10 (16.4%)	10 (30%)	7 (15%)

RT + adjuvant chemotherapy	7 (37%)	28 (46%)	14 (42%)	21 (45%)

Adjuvant chemotherapy	2 (11%)	7 (11%)	2 (6%)	7 (15%)

RT + Temozolomide	2 (11%)	8 (13%)	4 (12%)	6 (13%)

RT	1 (5.3%)	3 (5%)	1 (3%)	3 (6%)

RT + Lumostin	0 (0%)	1 (2%)	0 (0%)	1 (2%)

RT + IRESSA	0 (0%)	2 (3%)	2 (6%)	0 (0%)

Cortisone	0 (0%)	1 (2%)	1 (3%)	0 (0%)

Palliative therapy	0 (0%)	4 (7%)	1 (3%)	3 (6%)

### Phenotypic characterization of GBMs

All phenotypic characterizations of other factors examined for diagnostic purpose; p53, GFAP, MIB-index and mitosis in tissue sections were performed at the pathology department in our hospital. In patients that survived > 18 months, p53 mutations were found in 8 of 12 (67%) samples with moderate to high-grade HCMV-IEA infection as compared with 4 of 13 (31%) in patients with no or low-grade infection. p53 mutation was detected in 7 of 9 (78%) patients with moderate to high-grade HCMV-LA infection and in 5 of 16 (31%) with no or low-grade infection.

In patients with overall survival ≤ 18 months, p53 mutation was detected in 5 of 19 (26%) patients with moderate to high-grade HCMV-IEA infection and in 1/3 (30%) with no or low-grade infection. In patients with moderate to high-grade HCMV-LA infection, p53 mutation was detected in 5/11 (45%) of patients as compared to 1 of 11 (9%) patients with no or low-grade HCMV-LA infection.

While no association between p53 mutations was observed for HCMV IEA, we observed a significant association between HCMV late protein expression and p53 mutations (overall survival > 18 months; *p *.026) The extent of mitosis and MIB-index did not differ significantly between the two groups. All (n = 71) examined GBM specimens were positive for GFAP.

## Discussion

The etiology of GBM is unknown and the prognosis for GBM patients is still poor despite recent advances in medical treatment with temozolomide and radiotherapy [23,24]. In our study, we demonstrate a strong association between HCMV infection grade and overall survival. In the cohort of GBM patients that survived > 18 months, 40% had no or low-grade HCMV-IEA infection as compared with 8% of patients with overall survival ≤ 18 months. Young age (< 50 years) and good neurologic function have been reported to be important prognostic factors in GBM patients. In our study, we observed a significant association between no or low-grade HCMV-IEA infection (*p .0006*) and RPA subclass III-VI (*p < .0001*) and survival > 18 months. Further regression analyses of factors influencing survival of patients with low-grade versus high grade-HCMV infection in GBM tissues included, age, extent of resection, gender and RPA subclass. We found an odds ratio of 6.6 for low-grade HCMV infection and 8.2 for RPA subclass III and IV (*p .019 and p .002*, respectively). However, no association was found between survival and radical surgery, age, gender or gamma knife treatment (Table 2).

HCMV infection was detected exclusively in tumor cells and endothelial cells in the tumor part, but not in the non-tumor part of the tissue, which suggest that HCMV infection is restricted to the tumor cells. HCMV proteins may affect many central mechanisms in tumor biology and confer immune evasion mechanisms. For instance HCMV-IE72 and IE86 proteins interact with p53 and Rb that result in enhanced cellular proliferation [12,14]. In this study, we found that p53 mutation was associated with HCMV-LA expression (*p .026 *in patients surviving > 18 months), which implies a potential viral effect on p53. Interestingly, HCMV has been shown in vitro to cause mutations, in particular in p53 in cells that are transformed by IE72, IE86 and adenovirus E1A proteins [16]. These and other HCMV proteins also affect several additional pathways in the cellular machinery linked to tumor biology, such as cell cycle control, enhanced proliferation and migration of the cells, stimulation of telomerase activity, induced expression of COX-2 and 5-lipoxygenase and production of prostaglandin E2, leukotriene B4, and accumulated beta cathenin with potential key functions in HCMV induced oncogenesis or cancer progression [12,15-17,25-29]. In collaboration with Smit's group, we recently described an additional potential oncomodulatory role of HCMV US28, which is a viral G protein-coupled receptor encoded by HCMV (18). HCMV-US28 expression in the cells stimulated activation of STAT-3 and secretion of IL-6 and VGEF that led to enhanced proliferation and angiogenesis of HCMV infected cells. Interestingly, HCMV-US28 was found to be expressed in GBM tissue sections and GBM patients that had high grade phosphorylated STAT-3 (pSTAT-3) in their tumors had shorter time to tumor progression and overall survival (18). Interestingly, US28 expressing 3 T3-cells injected into nude mice formed tumors [29]. Smit's and Lira's groups established a transgenic mouse with US28 expressed in the intestine [29]. These animals developed adenomas and adenocarcinomas, further providing evidence that HCMV US28 may be oncogenic [29]. Furthermore, Soroceanu et al have recently demonstrated expression of US28 in 60% of GBM specimens and suggested that the invasive tumorigenic and angiogenic properties of US28 mediated by US28-CCL5 paracrine signaling may contribute to glioma progression [30].

A recent study by Dziurzynski et al showed that HCMV infection in glioblastoma stem cells (gCSCs) results in induction of viral IL-10 that activates HCMV-IE1 in monocytes and affects polarization of macrophages toward a M2 phenotype of macrophages. The authors claim that immunosuppressive M2 macrophages in GBM patients may contribute to gliomagenesis via induction of VEGF and enhanced angiogenesis, and increase immunosuppression by production of TGF-beta [31]. Both HCMV IL-10 and HCMV-US28 stimulate activation of STAT-3 (pSTAT-3) and thereby link them to tumorigenesis [32]. pSTAT-3 down regulates the anti-tumor response, suppresses macrophage activation, reduces NK cell and neutrophil cytotoxicity and impairs dendritic cell function [33].

In this study, we demonstrate that low-grade HCMV infection is strongly associated with long-term survival in glioblastoma patients, and hence the level of HCMV infection may provide a useful prognostic marker for survival of glioblastoma patients. This observation also implies that HCMV may be important in glioblastoma progression, and that the virus may provide a target for cancer therapy. In support of this hypothesis, we have recently demonstrated that the growth of HCMV infected human meduloblastoma xenografts can be inhibited by therapies targeting HCMV. Treatment of animals carrying HCMV positive human medulloblastoma xenografts with the anti-viral drug valganciclovir and the COX-2 inhibitor Celecoxib (that also targets HCMV replication) resulted in a 72% inhibition of tumor growth without using chemotherapy [20].

In summary, our results suggest that low grade HCMV infection in glioblastoma tumors is strongly associated with longer survival of GBM patients. This observation further supports the hypothesis that this virus may play a pathogenetic role in GBM tumors rather than representing an epiphenomenon. Targeting HCMV with anti-viral drugs may affect the viral load in the tumor and thereby prevent progressive disease. In support of this statement, we have recently completed the first clinical phase I/II trial on anti-viral therapy in glioblastoma patients, which indicate improved overall survival in patients receiving long-term treatment of valganciclovir.

## Competing interests

The authors declared no conflicts of interest. CS-N has served as a lecturer and in the Scientific Advisory Board for Roche and holds an investigational grant from the same company for a clinical study evaluating the effect of anti-viral treatment in GBM patients.

## Authors' contributions

CS-N designed and led the study, analyzed the data, and contributed to the manuscript writing. AR performed immunohistochemistry staining, in situ hybridization, analyzed tissue slides for immunohistochemistry, in situ hybridization and contributed to the manuscript writing. GS collected patient clinical data, analyzed RPA classification, and contributed to the manuscript writing. IP performed neurosurgical removal of GBM and collected patient clinical data. AO and JW confirmed the pathological diagnoses. AO analyzed tissue slides from immunohistochemistry. CT helped with immunohistochemistry staining. All authors approved the final version of the manuscript.

## References

[B1] Bergenheim T Registration on regional basis of patients with primary brain tumors. Regional differences disclosed Lakartidningen 2007 104 5 332 338 340-1 17328357

[B2] Lantos PL VS, KP Greenfield's neuropathology 1996 6 London: Arnold

[B3] Gupta M Djalilvand A Brat DJ Clarifying the diffuse gliomas: an update on the morphologic features and markers that discriminate oligodendroglioma from astrocytoma Am J Clin Pathol 2005 124 5 755 768 10.1309/6JNX4PA60TQ5U5VG 16203285

[B4] Affronti ML Overall survival of newly diagnosed glioblastoma patients receiving carmustine wafers followed by radiation and concurrent temozolomide plus rotational multiagent chemotherapy Cancer 2009 115 15 3501 3511 10.1002/cncr.24398 19514083

[B5] Cobbs CS Human cytomegalovirus infection and expression in human malignant glioma Cancer Res 2002 62 12 3347 3350 12067971

[B6] Mitchell DA Sensitive detection of human cytomegalovirus in tumors and peripheral blood of patients diagnosed with glioblastoma Neuro Oncol 2008 10 1 10 18 10.1215/15228517-2007-035 17951512 PMC2600830

[B7] Lucas KG The detection of CMV pp 65 and IE1 in glioblastoma multiforme J Neurooncol 2011 103 2 231 238 10.1007/s11060-010-0383-6 20820869

[B8] Taylor-Wiedeman J Monocytes are a major site of persistence of human cytomegalovirus in peripheral blood mononuclear cells J Gen Virol 1991 72 Pt 9 2059 2064 1654370 10.1099/0022-1317-72-9-2059

[B9] Soderberg-Naucler C Fish KN Nelson JA Reactivation of latent human cytomegalovirus by allogeneic stimulation of blood cells from healthy donors Cell 1997 91 1 119 126 10.1016/S0092-8674(01)80014-3 9335340

[B10] Sinclair J Human cytomegalovirus: Latency and reactivation in the myeloid lineage J Clin Virol: The Official Publication of the Pan American Society for Clinical Virology 2008 41 3 180 185 10.1016/j.jcv.2007.11.014 18164651

[B11] Cinatl J Jr Oncomodulatory signals by regulatory proteins encoded by human cytomegalovirus: a novel role for viral infection in tumor progression FEMS Microbiol Rev 2004 28 1 59 77 10.1016/j.femsre.2003.07.005 14975530

[B12] Boldogh I AbuBakar S Albrecht T Activation of proto-oncogenes: an immediate early event in human cytomegalovirus infection Science 1990 247 4942 561 564 10.1126/science.1689075 1689075

[B13] Bresnahan WA Albrecht T Thompson EA The cyclin E promoter is activated by human cytomegalovirus 86-kDa immediate early protein J Biol Chem 1998 273 34 22075 22082 10.1074/jbc.273.34.22075 9705351

[B14] Hagemeier C Functional interaction between the HCMV IE2 transactivator and the retinoblastoma protein EMBO J 1994 13 12 2897 2903 8026474 10.1002/j.1460-2075.1994.tb06584.x PMC395171

[B15] Muganda P Carrasco R Qian Q The human cytomegalovirus IE2 86 kDa protein elevates p53 levels and transactivates the p53 promoter in human fibroblasts Cell Mol Biol (Noisy-le-Grand) 1998 44 2 321 331 9593583

[B16] Shen Y Zhu H Shenk T Human cytomagalovirus IE1 and IE2 proteins are mutagenic and mediate "hit-and-run" oncogenic transformation in cooperation with the adenovirus E1A proteins Proc Natl Acad Sci USA 1997 94 7 3341 3345 10.1073/pnas.94.7.3341 9096395 PMC20371

[B17] Maussang D The human cytomegalovirus-encoded chemokine receptor US28 promotes angiogenesis and tumor formation via cyclooxygenase-2 Cancer Res 2009 69 7 2861 2869 10.1158/0008-5472.CAN-08-2487 19318580

[B18] Slinger E HCMV-encoded chemokine receptor US28 mediates proliferative signaling through the IL-6-STAT3 axis Sci Signal 2010 3 133 ra58 10.1126/scisignal.2001180 20682912

[B19] Maussang D Human cytomegalovirus-encoded chemokine receptor US28 promotes tumorigenesis Proc Natl Acad Sci USA 2006 103 35 13068 13073 10.1073/pnas.0604433103 16924106 PMC1559754

[B20] Baryawno N Detection of human cytomegalovirus in medulloblastomas reveals a potential therapeutic target J Clin Invest 2011 121 10 4043 4055 10.1172/JCI57147 21946257 PMC3195466

[B21] Mirimanoff RO Radiotherapy and temozolomide for newly diagnosed glioblastoma: recursive partitioning analysis of the EORTC 26981/22981-NCIC CE3 phase III randomized trial J Clin Oncol 2006 24 16 2563 2569 10.1200/JCO.2005.04.5963 16735709

[B22] Lachin JM Introduction to sample size determination and power analysis for clinical trials Control Clin Trials 1981 2 2 93 113 10.1016/0197-2456(81)90001-5 7273794

[B23] Stupp R Radiotherapy plus concomitant and adjuvant temozolomide for glioblastoma N Engl J Med 2005 352 10 987 996 10.1056/NEJMoa043330 15758009

[B24] Wen PY Kesari S Malignant gliomas in adults N Engl J Med 2008 359 5 492 507 10.1056/NEJMra0708126 18669428

[B25] Shen YH Human cytomegalovirus inhibits Akt-mediated eNOS activation through upregulating PTEN (phosphatase and tensin homolog deleted on chromosome 10) Cardiovasc Res 2006 69 2 502 511 10.1016/j.cardiores.2005.10.007 16316638

[B26] Straat K Activation of telomerase by human cytomegalovirus J Natl Cancer Inst 2009 101 7 488 497 10.1093/jnci/djp031 19318640

[B27] Qiu H Human CMV infection induces 5-lipoxygenase expression and leukotriene B4 production in vascular smooth muscle cells J Exp Med 2008 205 1 19 24 10.1084/jem.20070201 18180307 PMC2234367

[B28] Chen Z Degradation of p21cip1 in cells productively infected with human cytomegalovirus J Virol 2001 75 8 3613 3625 10.1128/JVI.75.8.3613-3625.2001 11264351 PMC114853

[B29] Bongers G The cytomegalovirus-encoded chemokine receptor US28 promotes intestinal neoplasia in transgenic mice J Clin Invest 2010 120 11 3969 3978 10.1172/JCI42563 20978345 PMC2964974

[B30] Soroceanu L Human Cytomegalovirus US28 Found in Glioblastoma Promotes an Invasive and Angiogenic Phenotype Cancer Res 2011 10.1158/0008-5472.CAN-11-0744 PMC3206211 21900396

[B31] Dziurzynski K Glioma-associated cytomegalovirus mediates subversion of the monocyte lineage to a tumor propagating phenotype Clin Canc Res: An Official Journal of the American Association for Cancer Research 2011 17 14 4642 4649 10.1158/1078-0432.CCR-11-0414 PMC3139801 21490182

[B32] Kortylewski M Inhibiting Stat3 signaling in the hematopoietic system elicits multicomponent antitumor immunity Nat Med 2005 11 12 1314 1321 10.1038/nm1325 16288283

[B33] O'Farrell AM IL-10 inhibits macrophage activation and proliferation by distinct signaling mechanisms: evidence for Stat3-dependent and -independent pathways EMBO J 1998 17 4 1006 1018 10.1093/emboj/17.4.1006 9463379 PMC1170450

